# Teaching communication skills in medical education: Best practice example of an interdisciplinary seminar in radiation oncology

**DOI:** 10.1007/s00066-023-02099-1

**Published:** 2023-06-12

**Authors:** Claudia Schmalz, Annette Rogge, Jürgen Dunst, David Krug, Katrin Liethmann

**Affiliations:** 1 Department of Radiation Oncology, University Hospital Schleswig-Holstein, Campus Kiel, Arnold-Heller-Str. 3, 24105 Kiel, Germany; 2 Nordseeklinik Helgoland, Helgoland, Germany Invasorenpfad 1040, 27498; 3 Department of Neurology, University Hospital Schleswig-Holstein, Campus Kiel, Kiel, Germany; 4 Psychooncology, Center for integrative Psychiatry ZiP gGmbH, Kiel, Germany

**Keywords:** Radiation oncology, Medical education, Counselling, Communication, Interdisciplinarity

## Abstract

**Background:**

Communication with patients is challenging, especially in radiation oncology. Therefore, radiation oncology is particularly suited to sensitize medical students for this topic and to train them competently. We report on experiences with an innovative teaching project for fourth- and fifth-year medical students.

**Materials and methods:**

The course, funded as an innovative teaching project by the medical faculty, was offered as an optional course for medical students in 2019 and again in 2022 after a pandemic-related break. The curriculum and evaluation form were developed through a two-stage Delphi process. The course consisted of, first, participation during counselling of patients prior to radiotherapy, mainly on topics with shared decision-making, and, second, a 1-week interdisciplinary block seminar with practical exercises. The topics covered a broad spectrum of the competence areas defined in the National Competence-Based Learning Objectives Catalog for Medicine (NKLM). The number of participants was limited to approximately 15 students because of the practical components.

**Results:**

So far, 30 students (all at least in the seventh semester or higher) have participated in the teaching project. The most frequent reasons for participation were the desire to acquire competence in breaking bad news and confidence in talking to patients. The overall evaluation of the course was very positive, with a grade of 1.08 + 0.28 (on a scale of 1 = totally agree to 5 = totally disagree) plus German grade 1 (very good) to 6 (very bad). Notably, participants’ expectations regarding specific competencies (e.g., breaking bad news) were also met.

**Conclusion:**

Although the evaluation results cannot be generalized to the entirety of medical students due to the limited number of voluntary participants, the very positive evaluation shows the need for such projects among students and can also be seen as an indication that radiation oncology as a patient-centered discipline is particularly well suited to teach medical communication.

## Introduction

In the future, communication with patients will play an increasingly important role in the everyday work of physicians [1, 10, 20]. In 2017, the German Master Plan for Medical Studies 2020 defined sound training in medical communication as one of the core objectives [21]. Implementation of the resulting high requirements formulated in the National Competence-Based Learning Objectives Catalogue for Medicine (NKLM) is to take place across the board in medical studies in Germany from 2025 [14].

Radiation oncology practice is accompanied by a variety of communicative challenges. In particular, communication ranges from technical/physical aspects of the therapy to giving a cancer diagnosis, guiding a patient through the course of radiotherapy, and accompanying them in end-of-life situations. All these occasions of conversation require good special knowledge and different skills. Beyond profound oncological knowledge, these consultations also demand essential psychological and emotional skills. Furthermore, the complex multimodal therapies and the presence of unclear fears and reservations regarding radiation treatment in many patients lead to extraordinary counselling situations. Radiation oncologists should therefore develop a special competence for such situations during their residency training and professional life. Thus, radiation oncology is particularly suited, both through the teaching physicians and the patients involved, to sensitize medical students to this topic and to train them competently [5].

Therefore, we have designed a communication seminar for fourth- to fifth-year medical students with the aim of introducing the experiences and competencies of radiation oncology into the training of physicians in the best possible way. We report on initial experiences and evaluation results after 1 year.

## Materials and methods

### Concept

The course was planned as an interdisciplinary project in 2018 and conducted for the first time in 2019 and again in 2022 after a pandemic-related break. The entire project was funded by the medical faculty as an innovative teaching project. The three initiators and leaders were two radiation oncologists, one of whom is a palliative care specialist, and a psycho-oncologist working in radiation oncology. Additional lecturers from other disciplines were invited to the seminar with reference to the requirements of the NKLM [3, 14].

The course was an optional elective course for fourth- and fifth year medical students. At this point, medical students have left the exclusively theoretical part of their studies. At the beginning of the herein presented course, all students participated in counselling of patients prior to radiation therapy. The subsequent main part of the course was a 1-week block seminar. The curriculum and evaluation questionnaire were developed using a two-stage Delphi process [4]. After written surveys of physician employees of different experience levels and specialties (*n* = 10, consisting of radiation oncologists *n* = 2, medical oncologists [both palliative care specialists] *n* = 2, general practitioner *n* = 1, radiologist *n* = 1, resident doctors *n* = 4) and joint discussion, those items that scored at least an average of 3.6 on a scale of 0 (disagree completely) to 4 (agree completely) were included in the curriculum. The topics covered a broad spectrum of the competency areas defined in the NKLM (Table 1). The number of student participants in the seminar was limited to approximately 15 participants because of the practical parts.

**Table 1:** Modules, content, didactics, learning goals, and their relation to the National Competence-Based Learning Objectives Catalog for Medicine (NKLM)

Module	Content	Didactics	Task/learning goal	Relation to the NKLM [14]
Participation in counselling of patient	Guided internship as observer, including debriefing	Lecture, discussion	Observe and analyze physician role model concerning content, role, and communication style	IV.2.3, A.‑F.01‑IIIc, A.‑F.03‑IIIc, A.‑F.04‑IIIc, A.‑F.08‑IIIc
Communication models	Theoretical basic models for communication, including constructivism	Lecture, exercise, discussion	Knowledge of basic communication theories and their relation to patient–physician encounters	VIII.3‑03.3.2, A.‑F.08‑IIIc, VIII.02-03.19
Augmentative and alternative communication	Theories and practice to enable communication when it is verbally restricted, e.g., via barriers in language, sensory perception, cognitive functions, age, etc.	Lecture, discussion, roleplay	Knowledge and use of communication tools	VIII.3‑04.2, III.8, IV.2.3, IV.2.4, VIII.3‑03
Presence and self-care: voice and breath	Theories and practice to handle voice, breath, and presence in general and within patient consultations, mindfulness, and self-care	Lecture, exercise, discussion	Deal with voice and breath within patient encounters	III.8, VIII.4‑04.5.2, VIII.5‑11.1.2, VIII.5‑11.1.11
Shared decision-making (SDM)	Theories and empiricism to models and practice of SDM, including guideline for own practice	Lecture, discussion, video-based roleplay and feedback	Understand SDM in theory and apply it in own practice	VIII.2‑02.6, VIII.2‑02.7
Understanding of roles	Theory of role models and changes in roles within the medical system	Lecture, exercise, discussion	Understand different ideas of physician role, apply for own role orientation	III.7, VIII.5‑01
Speaking/writing about patients	Ethical practice guideline, speaking/writing about patients, multi-professional collaboration	Lecture, exercise, discussion	Understand and apply respectful patient reporting	III.8, IV.2.3, IV.2.4, VIII.3‑03, A.‑F.09‑IIIc, VIII.5.11.1.11
Self-care and mindfulness	Theory and questionnaire to self-care as member of the medical system, especially RO	Lecture, self-awareness, discussion	Understand self-care, self-awareness of own resources and values	III.8, VIII.5‑11.1.2, VIII.5‑11.1.11, VIII.4‑04.5.2
Talking with children about their sick parents	Theories, empiricism, and practical insight into the work of an honorary initiative	Lecture, exercise, discussion	Understand and apply careful talking with children about their sick parents	III.8, IV.2.3, IV.2.4, VIII.3‑03, IV.2.6, V01.1.1.19
Medical ethics	Theories and discussion of values in clinical practice, role models	Lecture, discussion	Understand different ideas of physician role, apply for own role orientation	III.7, VIII.5‑01, III.8, IV.2.3, VIII.3‑03
Talking about emotions	Theoretical model and practical insight to deal with emotions of patients, relatives, and own emotions as physician	Lecture, discussion, roleplay	Understand and apply respectful handling of emotions	VIII.2‑03, A.‑F.08‑IIIc, VIII.2.03-19, VIII.5.11.1.11
Breaking bad news	Theories and empiricism to different models of communicating bad news to patients and their relatives	Lecture, discussion, roleplay	Understand and apply communication of bad news	VIII.2‑03, A.‑F.08‑IIIc, VIII.2.03-19, VIII.5.11.1.11
Processing mechanisms under (di)stress	Theories and empiricism to understand and deal with patients and relatives’ behavior after, e.g., bad news	Lecture, exercise, discussion	Understand information processing and its consequences for patient–physician encounters	VIII.2‑03, VIII.5.11.1.11
Psycho-oncology	Introduction into the workspace of psycho-oncology	Lecture, discussion	Understand role of psycho-oncology and know relevant offers of help	VIII.4‑01.3.1, VIII.5.11.1.11
Ethical case discussion	Theories and empiricism to decision-making in groups, ethical case discussion, tasks and goals of clinical ethics committees	Lecture, exercise, discussion	Understand and apply ethical case discussion	VIII.3‑03, VIII.6‑04.4.12

*III.7* Basic features of physician images and physician roles

*III.8* Tasks of the medical profession

*IV.2.3* The physician as a communicator

*IV.2.4* The physician as a member of a team

*IV.2.6* The physician as a responsible person

*A.‑F.01‑IIIc* Situationally appropriate performance of anamnesis and physical examination as well as structured summary of results

*A.‑F.03‑IIIc* Clinical decision-making

*A.‑F.04‑IIIc* Obtain informed consent for examinations and procedures in a patient-centered manner

*A.‑F.08‑IIIc* Structured information and counselling of patients

*A.‑F.09‑IIIc* Structured intra- and interprofessional handover of patients

*V01.1.1.19* Accompaniment of chronically ill patients and accompaniment of permanently ill patients

*VIII.2‑02.6* Shared decision-making process with patients and their relatives considering their circumstances

*VIII.2‑02.7* Organize and plan further diagnostics and treatments

*VIII.2‑03* The graduate reflects on typical sensitive topics in the everyday work of a physician and shapes his or her communication appropriately, even in emotionally challenging situations

*VIII.2‑03.19* Communicate truthfully and empathically with dying persons and their relatives

*VIII.3‑03* The graduate communicates adequately as a member of a team with representatives of different health care professions (…)

*VIII.3‑03.3.2* Apply communication models to de-escalate conflictual conversations within the team

*VIII.3‑04.2* Aspects of interprofessional health care and care for children and adults with intellectual or multiple disabilities, taking into account (…) communication characteristics

*VIII.4‑01.3.1* Including bio-psycho-social components into the encounter and counselling

*VIII.4‑04.5.2* Understand the importance of self-care as the basis of medical action (…), and know and apply individual measures to this end

*VIII.5‑1* The graduate will develop an understanding of the role of a physician

*VIII.5‑11.1.2* Apply basic knowledge of mindfulness methods (…) independently (…)

*VIII.5.11.1.11* Develop an increased awareness to meet patients and their concerns with the greatest possible mindfulness and presence

*VIII.6‑04.4.12* Explain the goals, tasks, and working methods of ethics consultation and ethics committees

The block seminar included the following topics: debriefing of the counselling participated in; communication models; augmentative and alternative communication; presence and self-care: voice and breath; shared decision-making (SDM); understanding of roles; speaking/writing about patients; self-care and mindfulness; talking with children about their sick parents; ethics in medicine; talking about emotions; breaking bad news; processing mechanisms under (di)stress; psycho-oncology; and clinical ethics. Content, didactics, learning goals, and relation to the NKLM are listed in Table 1.

### Interprofessional participation

The course unit “presence and self-care: voice and breath” was designed and taught by a trained opera singer and a performance artist, the course unit “augmentative and alternative communication” by a communication pedagogue, and the course unit “talking to children about their sick parents” by the leaders of a corresponding working group of the hospice initiative. The learning units “ethical case discussion” and “ethics in medicine” were newly introduced in 2022; these learning units were designed by a philosopher and a clinical ethicist [2].

### Practical exercises

In addition to participation in counselling of patients, approximately 50% of the sessions in the block seminar had a workshop character with practical exercises, roleplays, and discussions and methods of self-reflection [19]. Learning objectives of the practical exercises included improvement of self and role understanding, training of communication skills, and the topics of “breaking bad news,” “shared decision-making,” and decision discussions.

### Evaluation

Before the seminar began, students were surveyed regarding their motivation for participating in the communication seminar. In addition, the participants were asked to rate their own communication skills with patients and to indicate the extent to which it would be true to want to work with patients later in medical practice. There were two parts of evaluation: for “each module” [1], and at the end of the course for the whole course “overall” [2]. For both parts participants answered to a) criteria defined within the Delphi process on a five-point Likert scale (geometric equidistant points with two verbal poles: totally agree–totally disagree), b) German grade (1–6), and c) optional free-text comment. In addition, for each module, participants rated by self-report their achievement of learning objectives and their perceived usefulness with a five-point Likert scale (see above: totally disagree, totally agree). There was no knowledge test. Evaluation was pseudonymized.

## Results

### Participants

A total of 30 students (26 women, 4 men) participated in the teaching project, 13 of them in 2019 and 17 in 2022. The students were at least in their seventh semester of study; 12 had already completed a professional training. Only five of the participants indicated prior experience with communication seminars. All but one indicated a later aspiration to work as a physician with direct patient contact.

### Reasons for participation

**Fig. 1:**
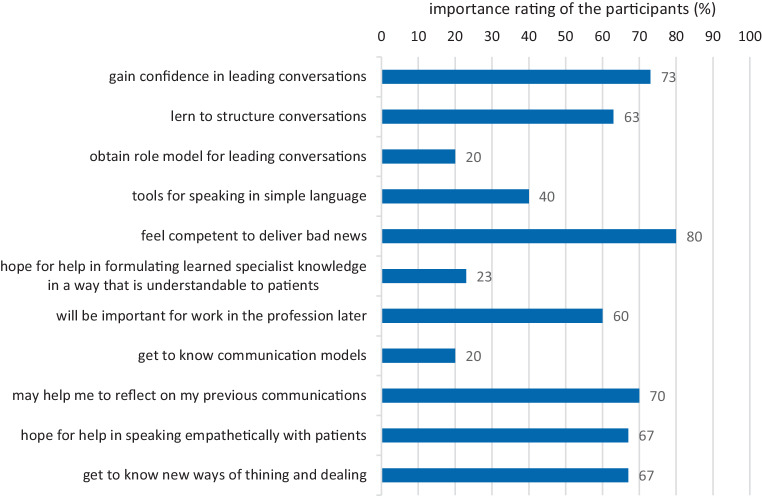
Motivation to attend the seminar

Participants rated their communication skills with patients as relatively good even before the seminar began (2.55 ± 0.55 on a scale of 1 = very good to 5 = poor). The most frequently cited reasons for attending the communication seminar were the desire to acquire better competence in breaking bad news and confidence in conducting conversations (Fig. 1).

### Evaluation results

Both evaluation parts, “each module” and “overall,” showed very positive results (Table 2 “overall,” Appendix: “each module”). In particular, the expectations formulated in advance regarding the acquisition of competencies for specific situations (e.g., breaking bad news) were met. In the free-text comments, the interprofessional participation and the practical exercises with roleplays were also specifically highlighted as instructive.

**Table 2:** Overall evaluation of the seminar. Mean values and standard deviation (SD)

On a scale of 1 (totally agree) to 5 (totally disagree), the entire seminar has	2019	2022
… provided me with new knowledge	1.23 (0.44)	1.18 (0.39)
… helped me to deepen my knowledge on the subject	1.15 (0.38)	1.0 (0)
… helped me to get to know new ways of thinking	1.31 (0.48)	1.0 (0)
… helped me to approach my future patients more empathetically	1.54 (0.78)	1.0 (0)
… helped me to feel more confident in talking to patients	1.54 (0.66)	1.06 (0.24)
… provided me with role models for conducting conversations	1.46 (0.66)	1.41 (0.61)
… helped me to formulate my acquired specialist knowledge in a way that is more understandable to patients	2.23 (1.36)	1.88 (0.86)
… interested me	1.0 (0)	1.0 (0)
Overall assessment of the seminar	1.08 (0.28)	1.06 (0.24)

## Discussion

Good communication with patients and active participation of patients in treatment decisions are playing an increasingly important role. In the US, radiation oncologists are also involved in the nationwide “choosing wisely” campaign with various questions [10]. This international initiative has developed brief recommendations for various specialities to reduce overuse and misuse. In Germany, a corresponding program for all clinics and departments has been established at the UKSH in Kiel as a national competence center for shared decision-making [8]. All physicians (> 90% of each clinic) underwent a multistage training program to improve communication for doctor–patient contacts. The scientific evaluation shows consistently positive effects, not only for patient satisfaction, but also in terms of cost efficiency [9]. The results confirm that communication skills in the medical field can be learned and taught on the one hand and will produce positive effects on the other hand [7].

Communication will become even more important in the medical profession in the future. Challenges for physicians not only concern communication with patients in an increasingly complex medical environment which is rapidly changing due to progress, but also the function as a member and leader of a multiprofessional team [12]. Therefore, communication skills have been integrated into the training curricula for medical students in many countries and will be given even greater consideration in medical studies as part of the implementation of the NKLM in Germany.

Radiation oncology is predestined to play an important role in these training segments. The seminar presented here was conducted by radiation oncologists with the support of a multiprofessional non-physician team and with the cooperation of radio-oncological patients. The very positive evaluation shows the need for such events among medical students on the one hand; on the other hand, our results can be seen as an indication that the topics based on radio-oncological everyday life, e.g., communication of complicated scientific facts and complex treatments, breaking bad news, addressing feelings, and being part of a multiprofessional team, are particularly well suited to teach communication. This is supported by a variety of studies about teaching projects in radiation oncology and probably also applies for digitally based learning [6, 13, 15–18, 22].

The course was an optional offer provided to medical students. The number of participants, however, had to be limited for organizational reasons. Therefore, the participants (about 5 to 10% of a study year) cannot be considered a representative sample, so that the evaluation results cannot necessarily be applied to the entirety of medical students. In addition, there might be a selection bias. Only motivated and interested students participated, which might cause a positive bias for evaluation. Moreover, it is also possible that the desire for face-to-face teaching after corona-related teaching restrictions positively influenced the evaluation results [11]. Nevertheless, in our opinion, such an event is certainly appropriate for teaching communication skills in future curricula. From our experience and considering the comments of the student participants, a group size of about 15 students seems to be optimal to ensure sufficient familiarity and supervision. Furthermore, repetition of the topic in the curriculum seems appropriate to increase the sustainability of the learning effect. In our opinion, the interprofessional participation chosen for this project is particularly well suited to best reflect the broad range of topics covered by the NKLM.

## Conclusion

Communication is one of the central competencies of physician action and activity. The findings of this innovative teaching project show that the field of radiation oncology is very well suited to teach this topic in medical school. Our goal with this seminar was to cover topics that are required in the NKLM but have been underrepresented in the medical curriculum so far. This represents a chance for radiation oncology to gain influence and establish a future role in shaping the minds of a future generation of doctors. We therefore hope that this course will be included in a future curriculum for medical students and may serve as a blueprint for innovative teaching in radiation oncology.

## Appendix

**Table 3:** Evaluation of each module of the seminar 2022, Median (Standard deviation; Number of answers)

On a scale of 1 (totally agree) to 5 (totally disagree), the module … has …	… provided me with new knowledge (1–5)	… helped me to deepen my knowledge on the subject (1–5)	… helped me to get to know new ways of thinking (1–5)	… helped me to approach my future patients more empathetically (1–5)	… helped me to feel more confident in talking to patients (1–5)	… provided me with role models for conducting conversations (1–5)	… helped me to formulate my acquired specialist knowledge in a way that is more understandable to patients (1–5)	… interested me (1–5)	Overall assessment of the seminar: German grade (1–6)	Perceived usefulness (1–5)
Communication models	2 (0.79; 17)	1.35 (0.49; 17)	2.12 (0.86; 17)	1.65 (0.79; 17)	2.47 (0.87; 17)	1.71 (0.85; 17)	3.41 (1.33; 17)	1.41 (0.51; 17)	1.76 (0.53; 17)	1.53 (0.51; 17)
Medical ethics	1.47 (0.51; 17)	1.24 (0.44; 17)	1.24 (0.44; 17)	2.12 (0.86; 17)	3.29 (0.69; 17)	3.18 (1.01; 17)	3.82 (0.81; 17)	1.18 (0.39; 17)	1.56 (0.50; 17)	1.65 (0.49; 17)
Presence and self-care: voice and breath	1.06 (0.24; 17)	1.29 (0.59; 17)	1 (0; 17)	1.94 (0.97; 17)	1.29 (0.59; 17)	3.29 (1.26; 17)	4.06 (1.25; 17)	1 (0; 17)	1.12 (0.33; 17)	1 (0; 17)
Shared decision-making (SDM)	1.88 (0.72; 16)	1.25 (0.45; 16)	1.94 (0.68; 16)	1.63 (0.81; 16)	1.81 (0.83; 16)	1.63 (0.81; 16)	3.06 (1.06; 16)	1.56 (0.63; 16)	1.47 (0.50; 16)	1.35 (0.61; 17)
Processing mechanisms under (di)stress	1.5 (0.63; 16)	1.31 (0.48; 16)	1.25 (0.45; 16)	1.25 (0.45; 16)	2.06 (0.68; 16)	2.31 (1.40; 16)	3.13 (1.15; 16)	1.13 (0.34; 16)	1.38 (0.50; 16)	1.53 (0.72; 17)
Talking about emotions	1.53 (0.62; 17)	1.06 (0.24; 17)	1.94 (0.90; 17)	1.18 (0.39; 17)	1.35 (0.49; 17)	2 (1.17; 17)	2.76 (1.20; 17)	1.06 (0.24; 17)	1.26 (0.44; 17)	1.29 (0.47; 17)
Understanding of roles	1.6 (0.74; 15)	1.33 (0.62; 15)	1.47 (0.64; 15)	2.5 (1.22; 14)	2.93 (1.22; 15)	2.4 (1.4; 15)	3.67 (1.18; 15)	1.07 (0.26; 15)	1.14 (0.36; 14)	1.13 (0.34; 16)
Speaking/writing about patients	1 (0; 16)	1.13 (0.34; 16)	1.75 (1.06; 16)	2.06 (1.44; 16)	1.94; (1.24; 16)	1.69 (0.87; 16)	2.25 (1.13; 16)	1.13 (0.34; 16)	1.22 (0.41; 16)	1.19 (0.40; 16)
Breaking bad news	1.76 (0.75; 17)	1.35 (0.49; 17)	1.65 (0.79; 17)	1.82 (0.95; 17)	1.94 (0.83; 17)	2.06 (1.03; 17)	2.88 (1.05; 17)	1.06 (0.24; 17)	1.35 (0.46; 17)	1 (0; 17)
Self-care and mindfulness	1.76 (0.97; 17)	1.41 (0.62; 17)	1.65 (0.86; 17)	3.18 (1.01; 17)	4 (0.94; 17)	4.18 (0.88; 17)	4.35 (0.70; 17)	1 (0; 17)	1.15 (0.34; 17)	1.06 (0.24; 17)
Psycho-oncology and ethical case discussion	1.29 (0.47; 17)	1.29 (0.69; 17)	1.41 (0.62; 17)	1.71 (0.69; 17)	3 (1; 17)	3 (1.22; 17)	3.65 (0.93; 17)	1.18 (0.39; 17)	1.24 (0.53; 17)	1.29 (0.59; 17)
Talking with children about their sick parents	1.13 (0.35; 15)	1.4 (0.63; 15)	1.27 (0.59; 15)	1.27 (0.80; 15)	1.87 (0.83; 15)	2.47 (1.13; 15)	2.53 (1.55; 15)	1.13 (0.35; 15)	1.2 (0.56; 15)	1.27 (0.59; 15)
Augmentative and alternative communication	1.4 (1.06; 15)	1.13 (0.35; 15)	1.4 (0.74; 15)	1.2 (0.41; 15)	1.73 (1.16; 15)	2.27 (1.22; 15)	1.33 (0.49; 15)	1.07 (0.26; 15)	1.4 (0.63; 15)	1.27 (0.59; 15)
Overall assessment at the end of the seminar	1.18 (0.39; 17)	1 (0; 17)	1 (0; 17)	1 (0; 17)	1.06 (0.24; 17)	1.41 (0.62; 17)	1.88 (0.86; 17)	1 (0; 17)	1.06 (0.24; 17)	
